# Patient-specific Hemodynamics of Severe Carotid Artery Stenosis Before and After Endarterectomy Examined by 4D Flow MRI

**DOI:** 10.1038/s41598-019-54543-x

**Published:** 2019-12-06

**Authors:** Seungbin Ko, Jeesoo Lee, Simon Song, Doosang Kim, Sang Hyung Lee, Jee-Hyun Cho

**Affiliations:** 1 Department of Mechanical Engineering, Hanyang University, Seoul, 04763 South Korea; 2 Institute of Nano Science and Technology, Hanyang University, Seoul, 04763 South Korea; 3 Department of Thoracic and Cardiovascular Surgery, Veterans Health Service Medical Center, Seoul, 05368 South Korea; 4 Department of Neurosurgery, SMG-SNU Boramae Medical Center, Seoul National University College of Medicine, Seoul, 07061 South Korea; 5 Bioimaging Research Team, Korea Basic Science Institute, Cheongju, 28119 South Korea

**Keywords:** Carotid artery disease, Atherosclerosis

## Abstract

Carotid endarterectomy (CEA) influences the carotid endoluminal anatomy, which results in hemodynamic changes before and after surgery. We investigated the hemodynamics of severe carotid artery stenosis before and after conventional endarterectomy with/without patch repair. An *in vitro* experiment utilizing carotid phantoms, which underwent a procedure that emulated CEA with/without the patch repair, was performed with a high-spatiotemporal resolution using 4D flow MRI. We evaluated an abnormal region of carotids, which consists of the normalized time-averaged wall shear stress (NTA|WSS|) and the oscillatory shear index (OSI), to account for continuous high-shear regions (high NTA|WSS| and low OSI) and chaotic low-shear regions, i.e., stenosis-prone regions (low NTA|WSS| and high OSI). The use of normalized hemodynamic parameters (e.g., NTA|WSS|) allowed comparison of diverse cases with different conditions of hemodynamics and vessel geometry. We observed that the stenosis-prone regions of the carotids with patches were noticeably larger than the corresponding regions in no-patch carotids. A large recirculating flow zone found in the stenosis-prone region of the internal carotid artery (ICA) of the postoperative carotids with patches partially blocks the flow path into ICA, and consequently the flow rate was not recovered after surgery unlike an expectation.

## Introduction

The relationship between pathophysiology of the vascular atherosclerosis and its vascular hemodynamic effects has been studied using various methods for decades^1–6^. Recently, studies have been conducted on the changes in the volumetric hemodynamics of the carotid artery resulting from endarterectomy with and without patch repair using computational fluid dynamics (CFD). Guerciotti, *et al*.^7^ suggested that the patch might lead to an unfavorable fluid dynamic condition, such as low and oscillating WSS. A similar result was derived by Harrison, *et al*.^8^, who revealed that a trimmed patch may allow a better hemodynamic benefit over a larger, full-size patch. Domanin, *et al*.^9^ explored long-term carotid restenosis between the two closure techniques and suggested that an excessively large widening of the carotid bulb should be avoided.

Time-resolved three-dimensional (3D) phase contrast magnetic resonance imaging (MRI), which is often called four-dimensional (4D) flow MRI, is a versatile flow measurement method that can simultaneously provide benchmark data for CFD verification and vessel geometry. The reproducibility of *in vivo* hemodynamic analyses using 4D flow MRI has been verified^10–12^, and can thus be utilized under complex hemodynamic conditions. Harloff, *et al*.^13^ investigated the influence of eversion carotid endarterectomy (CEA) on carotid hemodynamics using *in vivo* 4D flow MRI, and reported a significant decrease in the systolic WSS and oscillatory shear index (OSI) in the internal carotid artery (ICA) after the operation. However, to the best of our knowledge, the changes in volumetric carotid hemodynamics before and after conventional CEA and the comparison of the outcomes with and without patch repairs have seldom yet been investigated in detail using 4D flow MRI.

We investigated the volumetric hemodynamics of severe carotid artery stenosis before and after conventional endarterectomy comparing the two cases with and without patch repairs. *In vitro* experiments were planned to achieve a high spatio–temporal resolution. Two severe carotid artery stenosis patients were selected: one underwent patch repair CEA, and the other primary closure (i.e. without patch repair). Patient-specific, real-sized, solid carotid phantoms of each patient were fabricated with a 3D printer using computer tomography (CT) images before and after CEA. A custom-built, closed-loop flow circuit with a glycerin solution mixed in de-ionized water was used to emulate a patient-specific pulsatile blood flow. The pulsatile flow was measured with 4D flow MRI, and the volumetric velocity distribution and hemodynamic parameters, such as a combined parameter of WSS and OSI, were visualized and compared before and after CEA to assess the changes in hemodynamic characteristics.

## Methods

### Study population

**Figure 1:**
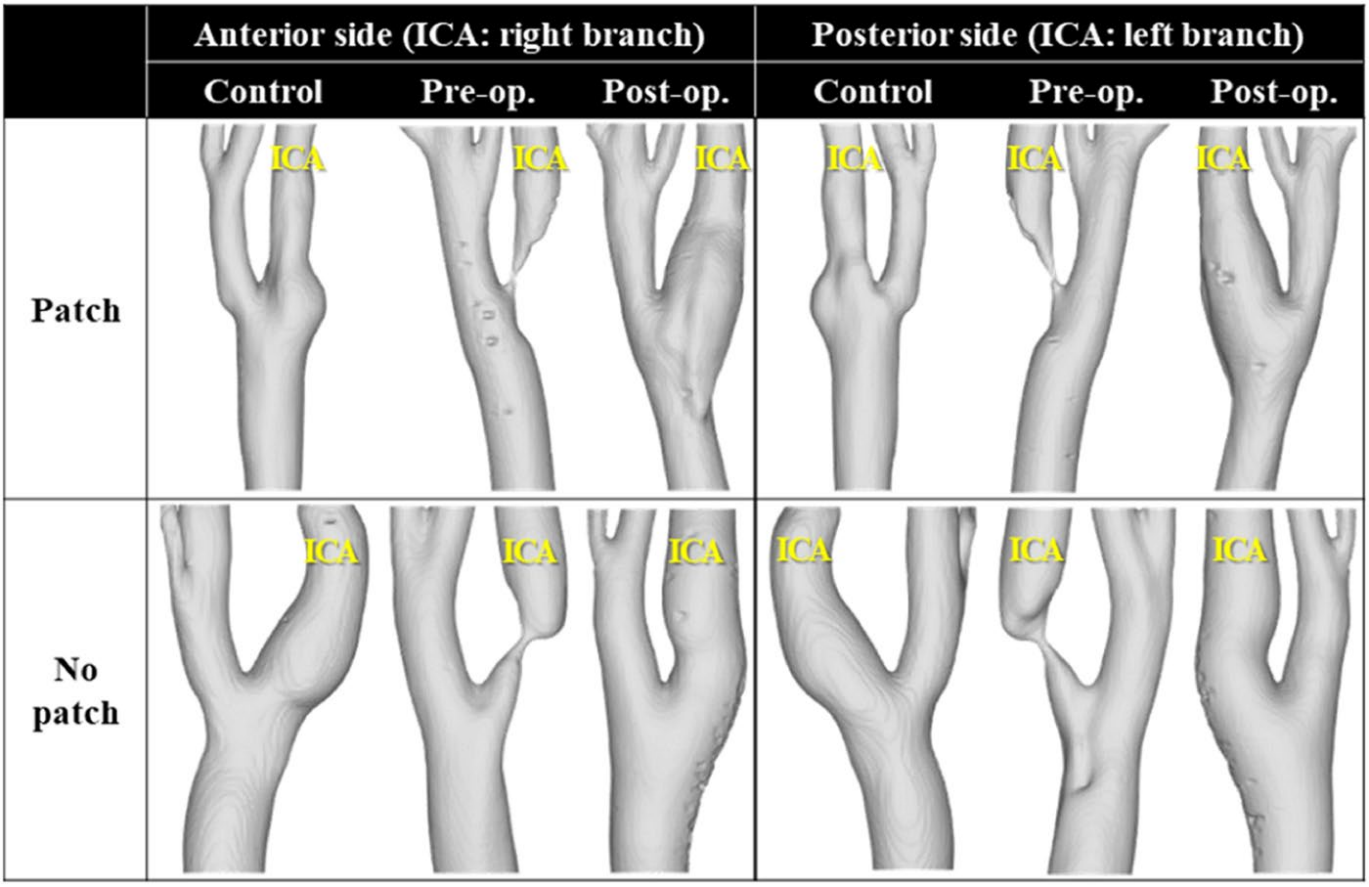
Reconstructed carotid geometries from 4D flow MRI measurements. The contralateral carotid was selected as the control for each patient.

Two carotid artery stenosis patients were retrospectively recruited among 10 candidates. A 68-year-old male patient underwent patch repair CEA in August 2014 and the other patient, a 70-year-old male patient, underwent CEA with primary closure in December 2014. The contralateral carotid arteries of each patient were selected as the control cases. Thus, six carotid models (pre-, post-, and contralateral carotid models per patient, and two patient sets) were included in this study (Fig. 1). The postoperative carotid CT images were obtained one week after surgery. The study was approved by an ethics committee of the Veterans Health Service Medical Center where the patients underwent surgery and was performed in accordance with the relevant guidelines and regulations. Informed consents for publication of identifying information in an online open-access publication were obtained from both participants.

### Experimental setup

Carotid phantoms for *in vitro* 4D flow MRI measurements were fabricated with a 3D printer using CT images as follows. The CT images stored in DICOM format files were loaded in MATLAB (R2017a, Mathworks, USA), and the carotid geometry was reconstructed using the isosurface function. The isosurface was exported as a stereolithography (STL) formatted file, and was trimmed smoothly using an STL editing program (Meshmixer v3.4.35, Autodesk, USA). Finally, the carotid phantoms were fabricated with a wall thickness of 1 mm using an STL-based 3D printer (Form 2, Formlabs, USA). A rigid and semi-transparent plastic resin was used as a test material, and the layer thickness (i.e., vertical increment) was 0.025 mm.

**Figure 2:**
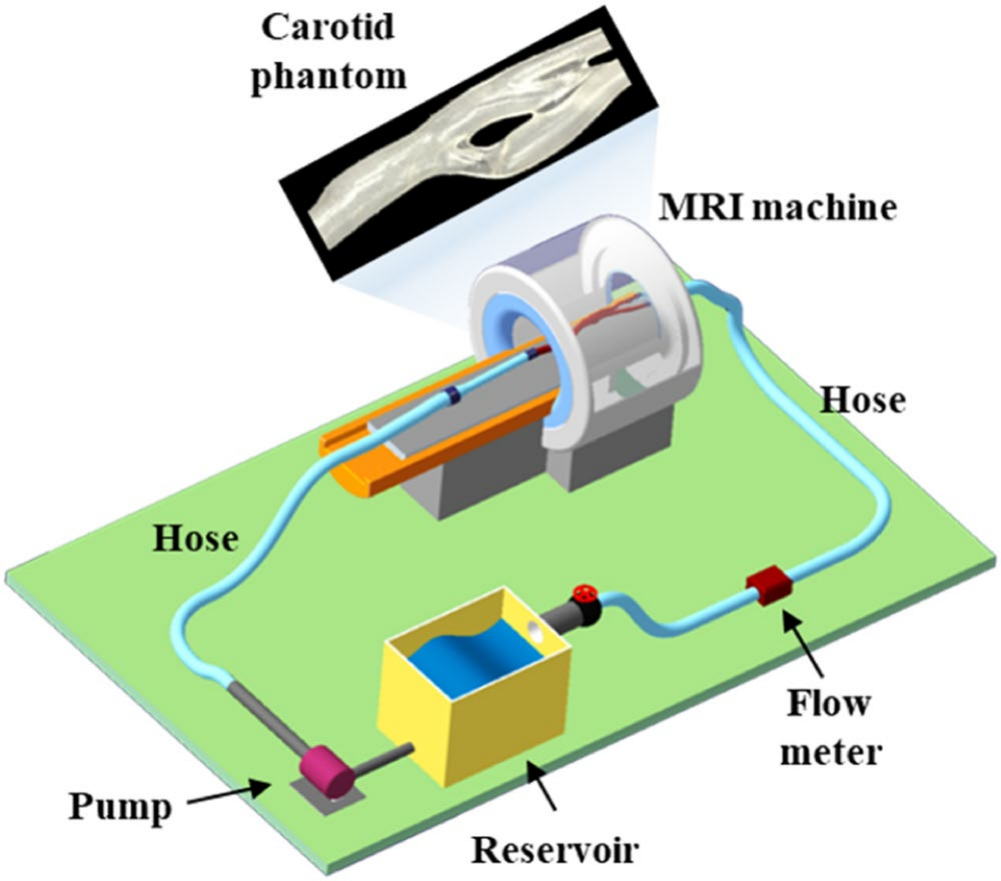
Schematic of the closed-loop flow circuit. Only the phantom part associated with the carotids was inside the bore of MRI machine to prevent magnetic field distortions.

A custom-made, closed-loop flow circuit was set to generate a patient-specific pulsatile flow, as shown in Fig. 2. The flow circuit consisted of a flow meter (IR–Opflow 1, Tecflow International, Netherlands), an acrylic reservoir, a microgear pump (GJ–N21.JF1S.SE, Micropump, USA), and a data acquisition instrument (NI USB–6361, National Instruments, USA) to simultaneously acquire real-time flow rate data and to deliver a patient-specific pulse form to the pump system. A urethane hose was used to connect the various components. The flow resistance in every downstream branch is set to be the same by connecting urethane hoses of the same inner diameter (4 mm) and the same length (1 m). Only the carotid phantom and part of the hose were inside the MRI bore to prevent magnetic field distortions. A 6 mmol/L copper sulfate aqueous solution was mixed with glycerin at a volume ratio of 3:2 (deionized water to glycerin), and the mixture was used as the working fluid with a viscosity and density similar to those of human blood (viscosity, 0.004 Pa·s; density, 1,100 kg/m^3^). The copper sulfate solution was utilized because it improves the signal-to-noise ratio of the MR signal^14,15^.

### 4D flow MRI measurements

The detailed conditions of the 4D flow MRI measurements are presented in Table 1. A 4.7 Tesla MRI machine (BioSpec 47/40, Bruker, Germany) installed at the Korea Basic Science Institute was used in the present study with a conventional birdcage radio frequency coil. The carotid position was adjusted to locate the bifurcation point as close as possible to the center of the field-of-view. The spatial resolution was 0.35 mm isotropic. The volumetric velocity data were measured by applying the balanced four-point velocity encoding method^16^ with the same encoding velocity value along each of the Cartesian axes. A trigger pulse was introduced to the MRI machine by the data acquisition instrument to apply prospective gating. Finally, a time-resolved, 3D, three-component (3C) velocity field was obtained by subtracting the magnetic resonance (MR) phase data acquired during pump-off from those acquired during pump-on to compensate for the intrinsic errors of MRI measurements arising from eddy currents or system drifts^14,17,18^.

**Table 1:** Conditions of 4D flow MRI measurements.

	Patch	No-patch
MRI machine	Bruker BioSpec 47/40	
Pulse sequence	FLOWMAP	
Scan mode	3D	
Zero-fill factor	2 for PE and SE direction	
Radio frequency coil	Birdcage coil	
Spatial resolution (mm)	0.35 (all directions)	
Temporal resolution (ms)	25	
Field-of-view (mm) (RO, PE, SE)	(44.8, 22.4, 22.4)	
Acquisition matrix (RO, PE, SE)	(128, 64, 64)	
Slice orientation (RO direction)	Sagittal (Foot-to-head)	
Flip angle (°)	30	
Repetition time (ms)	25	
Echo time (ms) (control/preoperative/postoperative)	2.2/2.25/2.25	2.2/2.75/2.75
Encoding velocity (cm/s) (control/preoperative/postoperative)	180/360/180	140/320/140
Scan time	Approximately 1 h 15 min	

The field-of-view of the phase encoding (PE) and slice encoding (SE) direction slightly varied depending on the carotid size. The zero-fill factor, which represents an amount of zero-filling in k-space, was set to two in the PE and SE directions to reduce scan time. A zero-fill factor of two means that only half of the k-space is acquired in that direction and the rest is filled with zeros. The RO refers to read-out direction.

### Hemodynamic parameters

We first evaluated two hemodynamic parameters, NTA|WSS| and OSI. The OSI indicates how often the WSS vector changes its direction during a cardiac cycle on the carotid surface. The NTA|WSS| is the averaged WSS over a cardiac cycle normalized by the dynamic pressure in the CCA, which is considered as the kinetic energy of the fluid flow. The adoption of NTA|WSS|, instead of WSS, allowed the comparison of present results with others reported in literature despite the different hemodynamic conditions of carotid flows.

We also defined an abnormal region in accordance to two conditions: a continuous high-shear region (NTA|WSS| > 0.25 and OSI < 0.05) and a chaotic low-shear region, i.e., stenosis-prone region (NTA|WSS| < 0.05 and OSI > 0.15). The threshold criteria of stenosis-prone region were determined based on the suggestion of Malek, *et al*.^3^. We visualized both abnormal regions simultaneously and examined the effects of the conventional CEA on the hemodynamics of the carotid artery that had been subjected to it. The detailed definition and calculation process of all hemodynamic parameters in the present study can be found in Supplemental Information.

## Results

### Abnormal region

**Figure 3:**
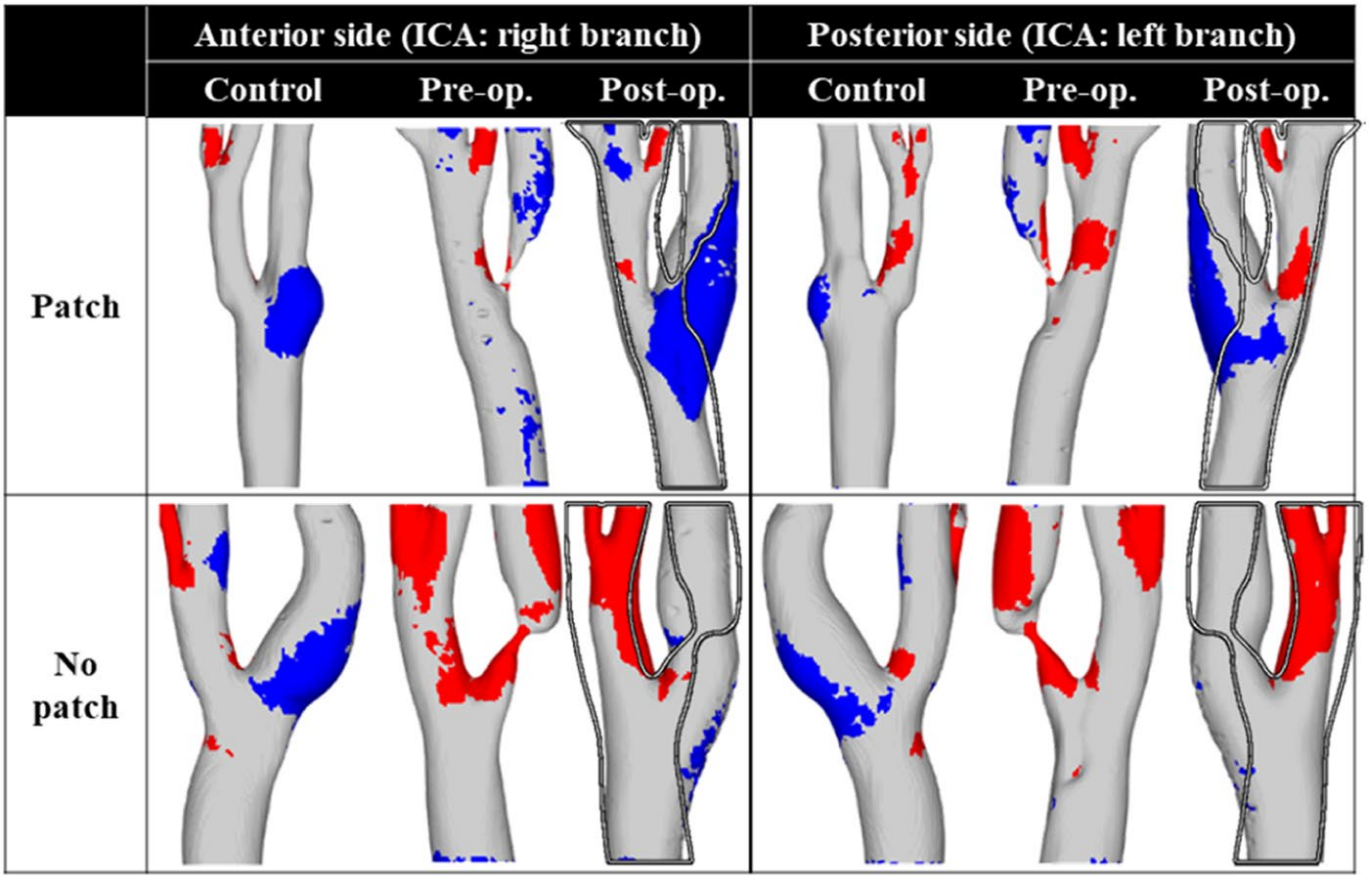
Contour plot of the abnormal region. The red region indicates a continuous high shear region (NTA|WSS| > 0.25 and OSI < 0.05), and the blue region shows a stenosis-prone region (NTA|WSS| < 0.05 and OSI > 0.15). The hollow line in the postoperative carotid cases represents an outline of the preoperative carotid shape.

After analyzing the flow data obtained by 4D flow MRI such as normalized time-averaged wall shear stress (NTA|WSS|) and OSI (see Supplemental Information), an abnormal region indicating a site prone to either the early development or rupture of atherosclerosis is shown in Fig. 3. The region highlighted in red shows a continuous high-shear region, while the region highlighted in blue indicates a chaotic low-shear region (or a stenosis-prone region). The hollow line in the postoperative cases delineates the preoperative carotid shape. This shows that the ICA bulb of the control and postoperative cases was mainly dominated by the chaotic low-shear in the patch-patient case. In particular, comparing the pre- and postoperative carotids, the chaotic low-shear region in the ICA bulb resulted from the recovery of the lumen following the CEA. Conversely, the continuous high-shear region in the ICA vanished after surgery.

**Figure 4:**
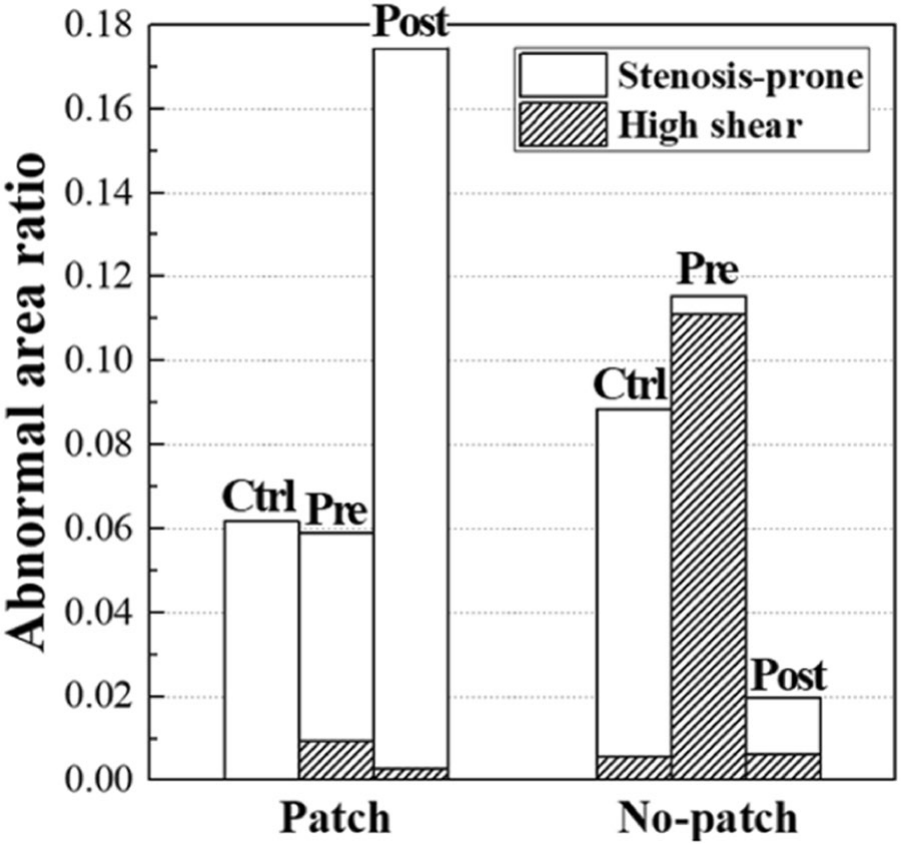
Area ratio of the abnormal region to the surface summation of internal carotid artery and common carotid artery.

For a quantitative analysis, the area ratio of the abnormal region to the surface summation of the ICA and common carotid artery (CCA) was calculated in Fig. 4. Interestingly, in the patch case, the ratio of the abnormal region of the postoperative carotid was nearly three times higher than that of the control, whereas it decreased to less than one-third in the no-patch case. Similar ratio changes were found in the carotids before and after CEA. On the other hand, the ratio of the continuous high-shear region was negligible in the postoperative carotids for both the patch and no-patch cases. It seems that the tapered narrowing of the ICA of the no-patch/preoperative carotid, compared to the patch/preoperative carotid case, yielded an increased ratio of 11% in the continuous high-shear region. The chaotic low-shear (stenosis-prone) region was responsible for most of the abnormal regions in both the control cases and the postoperative carotid case of the patient with the patch.

### Flow field and geometry of carotids

**Figure 5:**
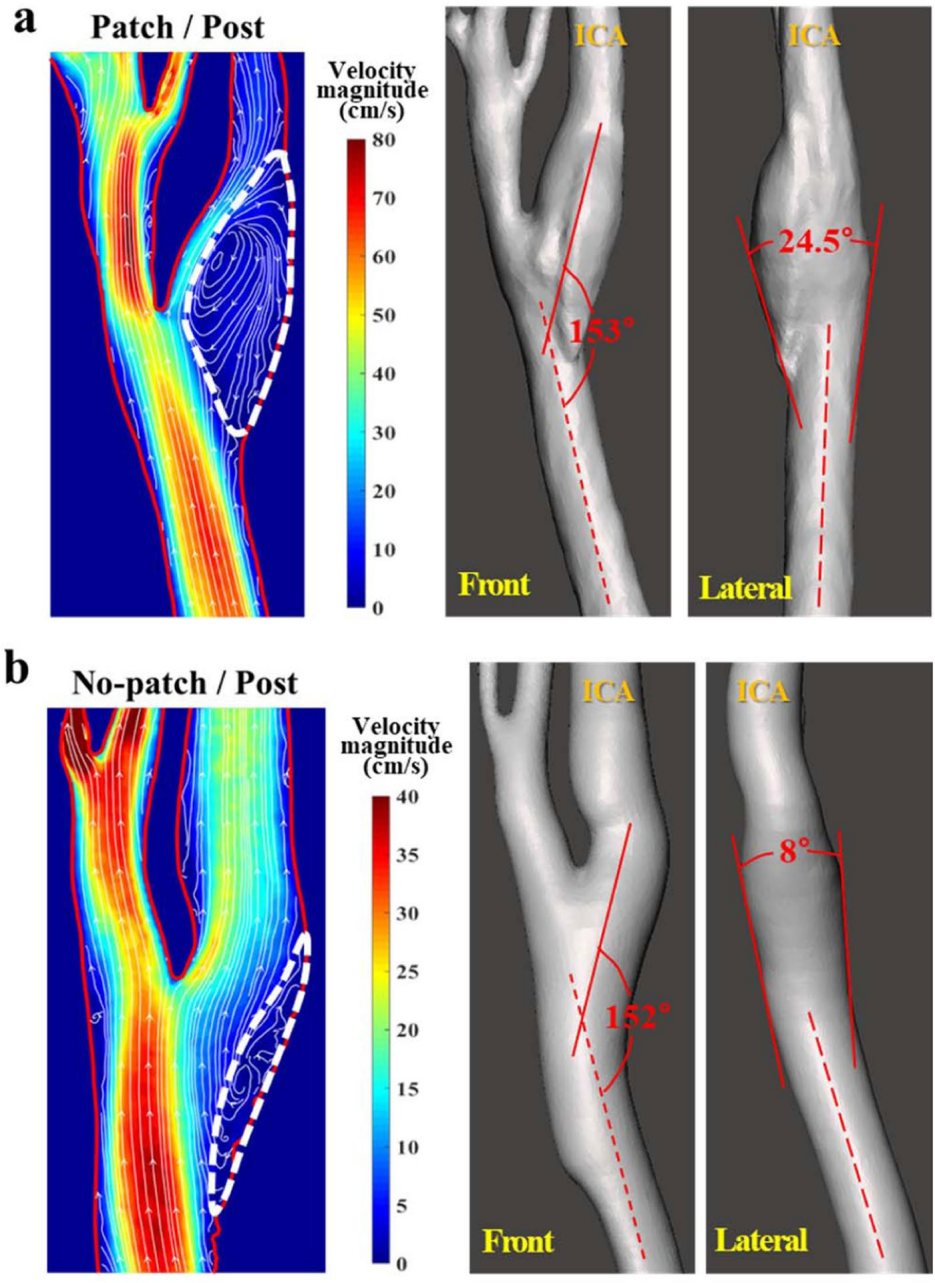
Streamlines at the peak flow rate and ICA–CCA spread angle in the (**a**) patch and (**b**) no-patch cases. Only postoperative carotids are presented for simplicity. The velocity magnitude contour is shown alongside the streamline plot. The recirculation zone of ICA is indicated with a white dotted line in the streamline plot.

To identify the cause of the abnormal region difference between the patch and no-patch cases, we examined the internal flow field and geometry of the carotids. In Fig. 5, a streamline plot with a velocity magnitude contour is shown on a cross-section of the postoperative carotids at the peak flow rate (t = 0.3 s in Fig. S1 of Supplemental Information). A flow recirculation zone (white dotted line) is clearly identified in the ICA bulb and it appears to be much greater in the patch/postoperative carotid than the no-patch/postoperative carotid. In fact, the volume of the recirculation zone in the patch/postoperative carotid was calculated to be approximately three times larger than that in the no-patch/postoperative carotid (patch/postoperative case: 0.601 mL and no-patch/postoperative case: 0.197 mL). This difference is attributed to the geometric differences between the two carotids. The ICA–CCA spread angles of the patch and no-patch carotid cases seem to be similar from the front view, but they differ noticeably from the perspective of the lateral view. Thus, it is reasonable to argue that the large recirculation zone in the patch/postoperative carotid is caused by the sudden volumetric expansion in the flow passage.

### Flow rate ratio of carotid branches

**Figure 6:**
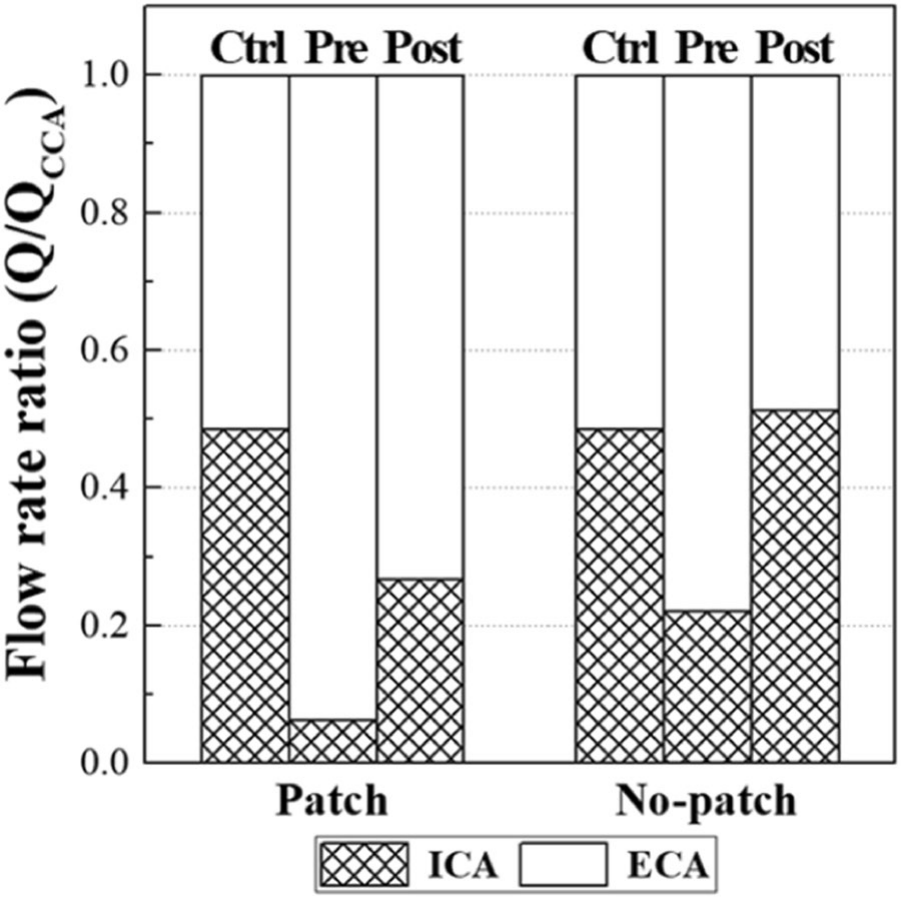
Flow rate ratio of carotid branches. The ratio is calculated by normalizing the mean flow rate of each carotid branch by the mean CCA flow rate.

The difference in the volume of the recirculation zone results in a noticeable difference in the flow rate in the ICA. Figure 6 shows the flow rate ratio of each carotid branch (ICA and external carotid artery (ECA)) to its own CCA. The ratio was calculated by dividing the average flow rate of each carotid branch by the CCA average flow rate. The control cases showed almost even flow rates between the ICA and ECA for both the patch and no-patch carotid cases. Obviously, the flow rates in the ICA for the preoperative carotids were much lower than those in the ECA owing to the stenotic lumen in both the patch and no-patch cases. However, the ICA flow rate recovered to a similar level to that of the ECA after CEA in the no-patch patient, whereas it showed only one-third of the ECA flow rate in the patch patient because of the large recirculation zone. This large recirculation zone in the ICA of the patch/postoperative carotid was found in the entire cardiac cycle, as shown in Fig. S3 of Supplemental Information. Its size varied from time to time, but it usually occupied the majority of the ICA bulb throughout the cardiac cycle.

## Discussion

*In vitro* experiments were conducted in the present study although *in vivo* studies are certainly more advantageous than *in vitro* studies in that they can explore actual physiological conditions. However, *in vivo* 4D flow MRI typically requires a long scan time, and is subject to a limited spatial resolution that ultimately reduces the WSS accuracy^19,20^. In particular, the WSS in vessels with a narrow lumen diameter, such as those characterized by stenoses, is severely affected by the spatial resolution. Since *in vitro* phantom studies are relatively free from scan time limitations, a higher resolution and a better WSS accuracy can thus be achieved^20^. We believe that it is advantageous to utilize a high-spatial resolution in *in vitro* studies to obtain accurate and precise hemodynamic parameters like WSS, and to observe the recirculation zone consisting of small-scale motions of fluid elements^21^.

The WSS and OSI criteria of the abnormal region have been determined by referring to various studies in the literature, but their findings are still under debate^7,8,22^. We think that the different cut-off values can be attributed to different flow conditions, characteristics of the study cohort, and the differences in the flow visualization tools (e.g., CFD versus 4D flow MRI). It is advantageous to compare the cut-off values in a nondimensional form by excluding the effects of such differences. Thus, we suggested the values of 0.05 for NTA|WSS| and 0.15 for OSI to classify the chaotic low-shear (stenosis-prone) region, while we used the values of 0.25 for NTA|WSS| and 0.05 for OSI to classify the continuous high-shear region. The threshold values for the stenosis-prone region were adopted from Malek, *et al*.^3^ mainly because they provided rich information required for the calculation of NTA|WSS|. Nevertheless, these cut-off values need to be confirmed to be statistically significant by precise, large-scale, and randomized clinical trials, so that they can be fully utilized in the surgeons’ diagnostic work.

It was found that the size of the recirculation zone in the patch and no-patch cases differed because it was affected by sudden changes (i.e., volume expansion in the ICA bulb) of the flow geometry of the carotids, as shown in Fig. 5. An ICA bulb that is too broad necessarily results in a large recirculation^23^, which may play a crucial role in the growth of the chaotic low-shear (stenosis-prone) region of low and oscillating WSS^2,24^. This is consistent with the previous findings of noticeable relationship between the ICA/CCA area ratio and the area of atherosclerosis-prone wall parameter^25^ or disturbed flow^22^. Recently, Domanin, *et al*.^9^ also confirmed that a larger expansion at ICA bulb is associated with a larger exposure to low WSS and consequently a higher tendency of remodeling into the optimal size of internal carotid arteries at 5 years after CEA. They suggested that the closure of carotid after CEA should avoid an excessively widening of the carotid bulb. However, it should be investigated thoroughly whether the excessive-widening results in restenosis over remodeling for optimal flow. Similarly, it should be emphasized that we are not questioning the utility of the patch itself, but that the presence of a recirculation zone in an abruptly widened ICA bulb may affect and inhibit the flow to ICA despite a successful CEA procedure. In addition, we are not suggesting that the recirculation should be eliminated completely. It is a common phenomenon in a branching or abruptly changing flow passage, and is often found in healthy carotids.

As described in the results section, the recirculation zone in the ICA bulb of the patch case acted as a flow disturbance (Fig. 6). In general, more blood flows to the ICA than to the ECA in humans. If a recirculation zone is large to block the passage of flow, as in the case of the patient with the patch repair in the present study, the ICA may not have enough blood flow throughout the entire cardiac cycle (Fig. S3 of Supplemental Information). Therefore, despite surgery, the dynamics of the blood flow may not be improved noticeably.

One of the limitations of the present study is that the study cohort was too small. Two patients were involved, which is a limited number to yield reproducibly significant results. Our ultimate goal is to obtain meaningful clinical insights based on detailed, quantitative experimentation data with a high spatio–temporal resolution by *in vitro* 4D flow MRI. Large-scale population studies with diverse cases, including healthy carotids, should be conducted in the future. The threshold values for NTA|WSS| and OSI may alter in the process of accumulating and analyzing flow data. In addition, CT images of the postoperative cases were obtained one week after surgery. Thus, our results reflect very early postoperative outcomes that resulted from an immediate change of hemodynamics after CEA. It is well known that the diameter of an artery adapts to both the acute and chronic changes in a hemodynamic condition, such as flow rate or WSS^26^. Follow-up data obtained 3 months after surgery should be analyzed to investigate how this adaptation takes place, and how it will eventually affect the long-term hemodynamics. In addition, it would be reasonable to consider changing the carotid phantoms to an elastic material that mimics the mechanical properties of the actual carotid vessel and to perform experiments considering robust fluid–structure interactions. Finally, conducting experiments with the flow division ratio obtained from a patient may help to obtain more realistic data because the WSS distribution is affected by the downstream flow resistance. It is also highly interesting to study how flow conditions affect the results of WSS distribution at the carotid bifurcation.

## Supplementary information

SUPPLEMENTARY MATERIAL

## Data Availability

The datasets generated during and/or analyzed during the current study are available from the corresponding authors on reasonable request.
