# Supplementary material for: Patient-specific Hemodynamics of Severe Carotid Artery Stenosis Before and After Endarterectomy Examined by 4D Flow MRI
Source: Sci Rep. 2019 Dec 6;9:18554. doi: 10.1038/s41598-019-54543-x (PMC6897954; doi:10.1038/s41598-019-54543-x)
Supplement: Supplementary file 1 — SUPPLEMENTARY MATERIAL [file 41598_2019_54543_MOESM1_ESM.docx]

**Patient-specific Hemodynamics of Severe Carotid Artery Stenosis
Before and After Endarterectomy Examined by 4D Flow MRI**

**SUPPLEMENTAL INFORMATION**

Seungbin Ko^1^, Jeesoo Lee^1,2,*^, Simon Song^1,2,*^, Doosang Kim^3,*^, Sang Hyung Lee^4^, Jee-Hyun Cho^5^

^1^Dept. of Mechanical Engineering, Hanyang University, Seoul, 04763, South Korea

^2^Institute of Nano Science and Technology, Hanyang University, Seoul, 04763, South Korea ^3^Dept. of Thoracic and Cardiovascular Surgery, Veterans Health Service Medical Center, Seoul, 05368, South Korea

^4^Dept. of Neurosurgery, SMG-SNU Boramae Medical Center, Seoul National University College of Medicine, Seoul, 07061, South Korea

^5^Bioimaging Research Team, Korea Basic Science Institute, Cheongju, 28119, South Korea

**Address for correspondence:**

Jeesoo Lee

Email: [jeesoolee@hanyang.ac.kr](mailto:jeesoolee@hanyang.ac.kr), Tel: +82–2–2220–4243, Fax: +82–2–2298–4634

Dept. of Mechanical Engineering, Hanyang University

222 Wangsimni-Ro, Seongdong-Gu, Seoul, 04763, South Korea

Doosang Kim

Email: [mdksr@paran.com](mailto:mdksr@paran.com), Tel: +82–10–6246–8606

Dept. of Thoracic and Cardio-vascular Surgery, Veterans Health Service Medical Center

53 Jinwhangdoro 61-Gil, Kandong-Gu, Seoul, 05368, South Korea

Simon Song

Email: [simonsong@hanyang.ac.kr](mailto:simonsong@hanyang.ac.kr), Tel: +82–2–2220–0423, Fax: +82–2–2298–4634

Dept. of Mechanical Engineering, Hanyang University

222 Wangsimni-Ro, Seongdong-Gu, Seoul, 04763, South Korea

**SUPPLEMENTARY METHODS**

**Details on study population**

The NASCET (North American Symptomatic Carotid Endarterectomy Trial) criteria scores for internal carotid artery (ICA) stenosis was more than 75% for the two carotid stenosis patients. The first patient (68-year-old male) suffered from insulin-dependent diabetes, hypertension, and bilateral carotid stenosis. The other patient (70-year-old male) suffered from diabetes, hyperlipidemia, a previous stroke episode, ischemic heart disease, and right severe carotid stenosis.

**Patient-specific flow rate**

A patient-specific pulsatile flow rate input is shown in Fig. S1. The flow rate was calculated from the velocity of each patient’s common carotid artery (CCA) measured by ultrasound Doppler. The periods of the cardiac cycle were 1.05 s and 0.85 s in the patch and no-patch cases, respectively. The periods of both the increasing and decreasing flow rate phases were approximately 0.2 s, and the maximum flow rate was measured at 0.3 s.

**Hemodynamic parameters**

The wall shear stress (WSS) represents the shear force exerted on the vessel wall, and it is defined as the multiplication of the fluid viscosity and the near-wall velocity gradient. Thus, it is important to precisely determine the wall boundary and the near-wall velocity for accurate estimation of the WSS. We calculated the WSS based on a velocity gradient correction method considering the partial volume effects^1^. Briefly, an isosurface of the vascular wall consisting of small triangular elements was defined following the thresholding of the MRI magnitude data in such a way to achieve the best division between the flow-only region (fluid) and the solid region (wall). A WSS vector ($\vec{\tau}$) was then calculated on the isosurface at each timeframe in accordance to the following equation,

$\vec{\tau}=\vec{F}_{s}-\left( \vec{F}_{s}\cdot\vec{n} \right)\vec{n}$, (1)

where $\vec{F}_{s}$ is the force vector defined on an isosurface element, and $\vec{n}$ is the unit normal vector of the element. The force vector in the Cartesian coordinate system can be expressed as

$\vec{F}_{s}=\mu\left[ \begin{matrix} 2\frac{\partial u}{\partial x} & \frac{\partial u}{\partial y}+\frac{\partial v}{\partial x} & \frac{\partial u}{\partial z}+\frac{\partial w}{\partial x} \\ \frac{\partial v}{\partial x}+\frac{\partial u}{\partial y} & 2\frac{\partial v}{\partial y} & \frac{\partial v}{\partial z}+\frac{\partial w}{\partial y} \\ \frac{\partial w}{\partial x}+\frac{\partial u}{\partial z} & \frac{\partial w}{\partial y}+\frac{\partial v}{\partial z} & 2\frac{\partial w}{\partial z} \end{matrix} \right]\cdot\vec{n}$, (2)

where $\mu$ is the dynamic viscosity of the fluid, and $u$, $v$, and $w$, correspond to the velocity components along the $x$, $y$, and $z$ directions of the Cartesian coordinate system, respectively. The velocity gradient terms were obtained by locating the exact intravoxel wall position by considering the partial volume effects and assuming a linear near-wall velocity profile.

The magnitude of the time-resolved WSS vector can be averaged over a cardiac cycle on an isosurface element as follows,

$TA\left| WSS \right|=\frac{1}{T}\int_{0}^{T} \left| \vec{\tau} \right|dt$, (3)

where T is the period of the cardiac cycle. This equation takes into account the temporal variation of the magnitude of the WSS vector. The TA|WSS| value should be normalized by the dynamic pressure for quantitative comparisons of the before and after carotid endarterectomy (CEA), or the repair and no-repair cases. The dynamic pressure, which has the same unit as the WSS, can be considered as the kinetic energy of the fluid flow and is defined as^2^

$Dynamic pressure=\frac{1}{2}\rho\bar{V}^{2}$, (4)

where $\rho$ is the density of the fluid, and $\bar{V}$ is the characteristic velocity, which was set to an average CCA velocity of 12 mm upstream of the bifurcation. Note that the average CCA velocity is defined by the average flow rate over a cardiac cycle divided by the cross-sectional area of CCA. The normalized TA|WSS| is denoted as NTA|WSS| hereafter.

The oscillatory shear index (OSI) indicates how often the WSS vector changes its direction during a cardiac cycle on an isosurface element, and is defined as^3^

$OSI=\frac{1}{2}\left( 1-\frac{\left| \int_{0}^{T} \vec{\tau}dt \right|}{\int_{0}^{T} \left| \vec{\tau} \right|dt} \right)$. (6)

The OSI ranges from 0 to 0.5. Zero OSI means that there is no change in its direction during a cardiac cycle, whereas 0.5 indicates a complete reversal of the original direction.

Referring to either the NTA|WSS| or OSI may be limited by the fact that the two parameters uncouple the changes of the WSS magnitude and direction during a cardiac cycle. To consider the two essential hemodynamic wall parameters simultaneously in a region with abnormal hemodynamic characteristics, we define an abnormal region in accordance to two conditions: a continuous high-shear region, and a chaotic low-shear region (or stenosis-prone region). The former is defined based on the condition that NTA|WSS| > 0.25 and OSI < 0.05, while the latter indicates a region where NTA|WSS| < 0.05 and OSI > 0.15. These stenosis-prone region criteria were determined following the studies of Malek*, et al.*^4^ and Harrison*, et al.*^5^. Harrison*, et al.*^5^ investigated the hemodynamics of the carotids after CEA using numerical simulations. Correspondingly, we assumed a CCA diameter of 7.2 mm to calculate the dynamic pressure. A continuous high-shear region is known to cause a rupture of vulnerable atherosclerotic plaques^6,7^. Conversely, a frequently oscillating WSS of a low magnitude indicates a chaotic and a weak flow, and is responsible for the early development of atherosclerosis manifested as intimal hyperplasia^8,9^. We visualized both regions simultaneously and examined the effects of the conventional CEA on the hemodynamics of the carotid artery that had been subjected to it. The hemodynamic parameters were calculated using custom-built MATLAB code. The 4D flow MRI data were partially cut before calculation to exclude the outliers near the two ends of the field-of-view. In addition, a divergence-free smoothing filter for a wall-bounded flow was applied to the velocity data before calculation for accurate estimation of WSS by satisfying the mass conservation law^10^.

**SUPPLEMENTARY RESULTS**

**Distributions of WSS and OSI**

The normalized average wall shear stress (NTA|WSS|) on the carotid surface is shown in Fig. S2a. Despite the different flow conditions of the patch and no-patch cases, a single, common legend is used owing to the applied normalization. Both cases yielded high NTA|WSS| values in the internal carotid artery (ICA) that was nearly obstructed before the operation (black arrow), and low NTA|WSS| values after the surgical mitigation of the stenosis. An increased local NTA|WSS| value was observed at the outer part of the distal ICA in the no-patch/preoperative case, which was caused by a strong jet flow that impinged on the vessel wall through the stenotic region. The impinging site as well as the corresponding continuous high-shear region disappeared after the carotid endarterectomy. Interestingly, another local region with increased NTA|WSS| values in the ICA was developed after surgery in both cases. As indicated by the white arrows of the “Postoperative” contours in Fig. S2a, the large ICA bulb contained a spot directly adjacent to the bifurcation apex with a high NTA|WSS| value, implying that a complicated hemodynamic behavior may occur. In addition, the NTA|WSS| of the patch/postoperative ICA were lower than those of the no-patch/postoperative ICA. However, the flow geometries of the proximal ICA appeared to be similar, thus indicating that a detailed hemodynamic analysis is required.

The OSI distribution in Fig. S2b was observed to be generally opposite to the NTA|WSS| distribution. That is, a region with high NTA|WSS| values, such as the stenotic ICA or the jet impinging region in the no-patch/postoperative case (black arrow), displayed a low OSI. Conversely, the ICA bulb was dominant with a high OSI value after surgery, which is similar to the control cases. Most of the high OSI values were observed at the ICA or CCA, and the flow in the external carotid artery (ECA) rarely changed its direction during the cardiac cycle in all the studied cases.

**SUPPLEMENTARY REFERENCES**

1. Ko, S., Yang, B., Lee, J., Song, S. & Cho, J. H. *Improvement of wall shear stress estimation accuracy by considering partial volume effec of 4D flow MRI in relation to a relative resolution* (preparing, 2019).

2. White, F. M. *Fluid Mechanics*. 7th edn (McGraw Hill, 2011).

3. He, X. & Ku, D. N. Pulsatile Flow in the Human Left Coronary Artery Bifurcation: Average Conditions. *J. Biomech. Eng.-Trans. ASME* **118**, 74-82 (1996).

4. Malek, A. M., Alper, S. L. & Izumo, S. Hemodynamic shear stress and its role in atherosclerosis. *JAMA* **282**, 2035-2042 (1999).

5. Harrison, G. J. *et al.* Closure technique after carotid endarterectomy influences local hemodynamics. *J. Vasc. Surg.* **60**, 418-427 (2014).

6. Tang, D. *et al.* Sites of rupture in human atherosclerotic carotid plaques are associated with high structural stresses: an in vivo MRI-based 3D fluid-structure interaction study. *Stroke* **40**, 3258-3263 (2009).

7. Slager, C. J. *et al.* The role of shear stress in the destabilization of vulnerable plaques and related therapeutic implications. *Nat. Clin. Pract. Cardiovasc. Med.* **2**, 456-464 (2005).

8. Dhawan, S. S. *et al.* Shear stress and plaque development. *Expert Rev. Cardiovasc. Ther.* **8**, 545-556 (2010).

9. Peiffer, V., Sherwin, S. J. & Weinberg, P. D. Does low and oscillatory wall shear stress correlate spatially with early atherosclerosis? A systematic review. *Cardiovasc. Res.* **99**, 242-250 (2013).

10. Im, C., Ko, S., Lee, J. & Song, S. *Divergence-free smoothing for denoising 4D flow MRI measurements of a wall-bounded flow* (preparing, 2019).


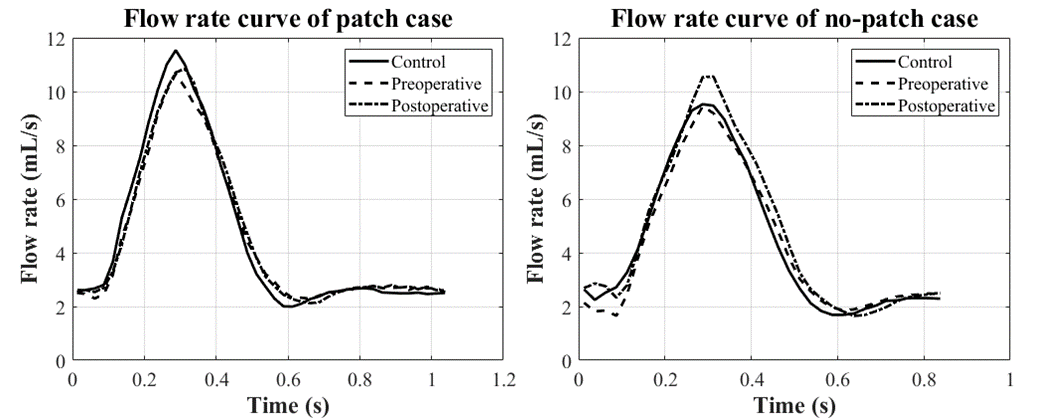
**Figure S1.** Patient-specific pulsatile flow rate input for in vitro experiments. The flow rate was calculated from each patient’s blood flow velocity of common carotid artery measured by ultrasound Doppler. The cardiac cycle periods were 1.05 s and 0.85 s for the patch and no-patch cases, respectively.

**
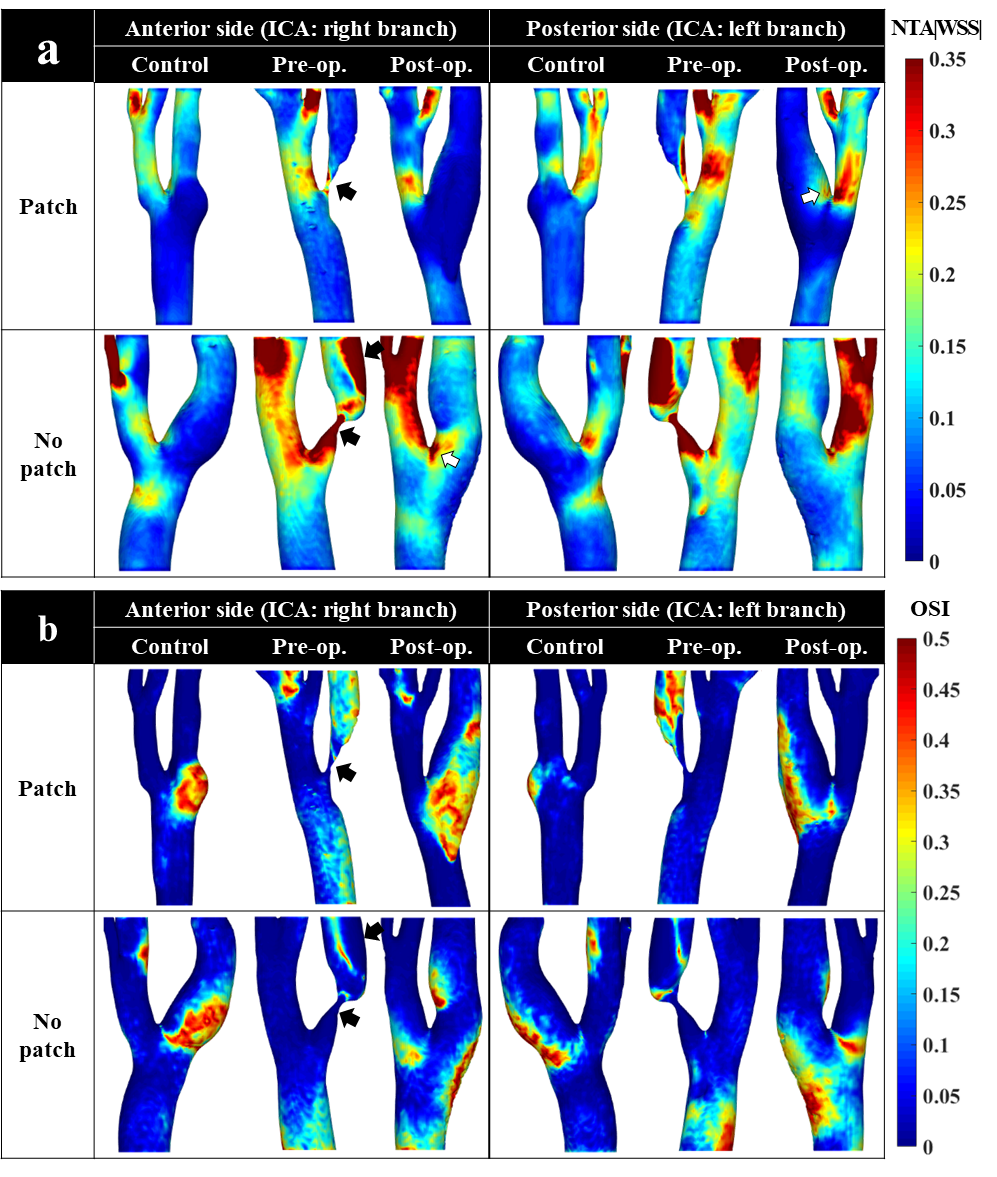
Figure S2.** (a) NTA|WSS| and (b) OSI contour plot. The solid black arrows indicate a region of high NTA|WSS| and low OSI in ICA. The white arrows indicate a region with an unexpectedly high WSS in a postoperative carotid cases.


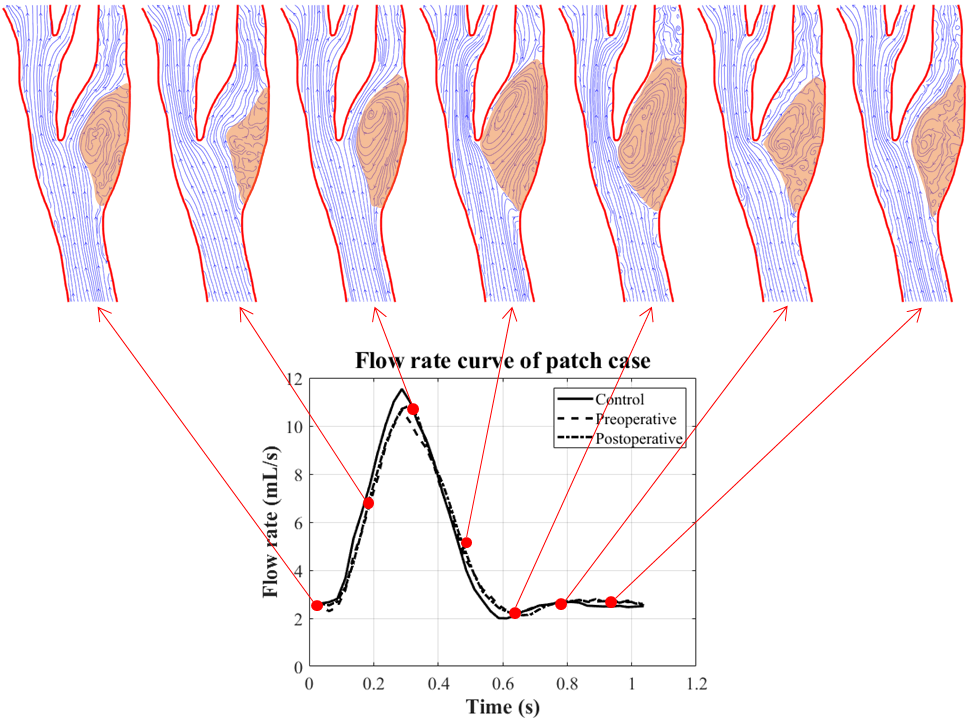
**Figure S3.** Streamline of patch/postoperative carotid for the entire cardiac cycle. The separation bubble is highlighted as an orange shaded region.
